# Ketamine Differentially Attenuates Alcohol Intake in Male Versus Female Alcohol Preferring (P) Rats

**DOI:** 10.4303/jdar/236030

**Published:** 2017-07-28

**Authors:** Amir H. Rezvani, Edward D. Levin, Marty Cauley, Bruk Getachew, Yousef Tizabi

**Affiliations:** 1 Department of Psychiatry and Behavioral Sciences, Duke University Medical Center, Durham, NC 27710, USA; 2 Department of Pharmacology, College of Medicine, Howard University, Washington, DC 20059, USA

**Keywords:** alcohol intake, alcohol preference, alcohol use disorders, glutamate receptors, NMDA receptor, sex differences

## Abstract

**Background:**

Although various pharmacological tools in combating addiction to alcohol are available, their efficacy is limited. Hence, there is a critical need for development of more effective medications. Recent advances in the field have identified the glutamatergic system as a potential novel target for intervention in addictive behaviors.

**Purpose:**

Hence, we evaluated the effects of acute administration of low (subanesthetic) doses of ketamine, an NMDA receptor antagonist, on alcohol intake and alcohol preference in both male and female rats.

**Study design:**

Adult alcohol preferring (P) rats were exposed to two-bottle choice (ethanol 10% and water) for at least three weeks following a nine-day training period and the effects of various doses of ketamine (5 mg/kg, 7.5 mg/kg, and 10 mg/kg, injected subcutaneously, SC) on consumption of alcohol over various time periods during a 24 h interval were measured.

**Results:**

Our results indicate that ketamine treatment significantly reduced both alcohol intake and preference in a time- and dose-dependent manner in both sexes. Moreover, a differential sensitivity between the sexes was observed. Thus, although alcohol intake was higher in males, female rats responded much more strongly to the highest dose of ketamine than males in the initial time periods.

**Conclusion:**

It is concluded that glutamatergic receptor manipulations may be of therapeutic potential in addiction to alcohol and that different sexes may respond differentially to such treatments.

## 1. Introduction

Current pharmacological tools in combating addiction to alcoholism or alcohol use disorders (AUDs) include disulfiram, an acetaldehyde dehydrogenase inhibitor that induces aversion; naltrexone, an opioid antagonist that blunts the rewarding effects of alcohol; and acamprosate, a synthetic gamma-aminobutyric acid (GABA) analog that may act as a functional N-methyl-D-Aspartate (NMDA) receptor antagonist [1,2]. All these drugs are modestly effective and in a relatively small number of patients [3,4]. Hence, more effective treatments are urgently needed.

It has become apparent that no single drug can help all individuals addicted to alcohol. A greater diversity of effective treatments will help with the tailoring of the most effective therapy to the needs of the diverse array of alcoholics. Although progress in this field has been hampered by the neurobiological complexity and lack of complete understanding of the crucial circuitries, neurotransmitters and/or receptor systems, diligent research has identified some potential targets. One of these targets is the neuronal glutamatergic system.

Glutamate is the major excitatory neurotransmitter in the brain with a number of critical functions that are primarily mediated through its interactions with two distinct classes of receptors: the ionotropic receptors, which are ligand-gated ion channels, and are further classified as NMDA, alpha-amino-3-hydroxy-5-methylisoxazole-4-propionic acid (AMPA), and kainate receptors, based on their sensitivity to selective agonists. The other class is the metabotropic glutamate receptors (mGluRs), which are G-protein-coupled and consist of at least eight different subtypes [5]. Of relevance to this study is the fact that both the ventral tegmental area (VTA) and nucleus accumbens (NAcc), which are directly associated with reward, receive extensive glutamatergic inputs [6]. Indeed, numerous studies have verified a strong interaction between alcohol and the glutamatergic system and applicability of this system in development of novel therapeutics in countering alcoholism [6, 7, 8]. Hence, the main purpose of this study was to elucidate the role of ionotropic glutamate receptors, particularly the NMDA receptors in alcohol intake, using an animal model. We used low, subanesthetic and nonsedative doses of ketamine, an NMDA receptor antagonist, in our studies. Moreover, because of differential response of the two sexes to drugs of abuse and the treatment modalities [9], we evaluated the effects of this drug in both male and female rats.

## 2. Methods

### 2.1. Animals

Adult male (*N* = 8) and female (*N* = 10) selectively-bred alcohol preferring (P) rats (12–14 weeks old) obtained from a breeding colony maintained at Duke University were used [10, 11]. All animals were housed individually in a standard laboratory with controlled temperature of 21 *±* 2 °C and humidity of 50*±*10 percent and a 12:12 h reversed light cycle (lights off: 07:00–19:00). The P rats were initially trained for alcohol drinking by exposing them to one bottle choice containing 8% alcohol (three consecutive days), then 9% alcohol (three consecutive days) and then 10% alcohol (three consecutive days). Following this training, they were given a free choice between water and a solution of 10% (v/v) alcohol for the remainder of the study [10, 11]. Rats were housed individually in cages that were fitted with two 100-mL graduated Richter drinking tubes for the recording of water and alcohol intake. These rats were given ad libitum access to food (5001 Rodent Chow; Lab Diet, Brentwood, MO, USA), water and alcohol at all times. Rats were on alcohol and water for at least three weeks before the drug treatment began. All studies were performed in accordance with the regulations outlined by the Animal Care and Use Committee of Duke University.

### 2.2. Preparation of drugs

Alcohol solutions were prepared twice a week from 100% reagent grade 200 proof pure ethyl alcohol (Koptec, King of Prussia, PA, USA) and tap water. Ketamine solution in a concentration of 10 mg/mL was purchased from Henry Schein (Melville, NY, USA) and was diluted with saline to obtain different concentrations.

### 2.3. Experimental protocol

The following experiments were performed to study the acute effects of ketamine on alcohol intake in both male and female alcohol preferring (P) rats.

After the P rats exhibited a stable baseline for intake of 10% alcohol solution and water for three consecutive weeks, they were injected subcutaneously (SC) with 5.0 mg/kg, 7.5 mg/kg, and 10.0 mg/kg ketamine or an equal volume of saline in a cross-over design with a counterbalanced order of doses. The interval between injections was at least three days. The volume of injection was 1 mL/kg. All injections were given between 9 AM and 10 AM and 15 min before exposure to alcohol. Alcohol and water intakes were recorded at 2 h, 4 h, 6 h, and 24 h after each treatment. Alcohol consumption is reported as grams of alcohol consumed per kg body weight per hour (g/kg/h). Alcohol preference is reported as volume of alcohol consumed divided by total fluid consumed (alcohol and water) during the specific period (% alcohol preference).

### 2.4. Statistical analysis

The data were assessed with the analysis of variance for repeated measures. Significant interactions were followed up by tests of the simple main effects. Planned comparisons were made between performance in control condition and each of the ketamine doses. The cut-off for statistical significance was *P* < .05, two-tailed.

## 3. Results

### 3.1. Ketamine effects on alcohol consumption and preference

**Figure 1:**
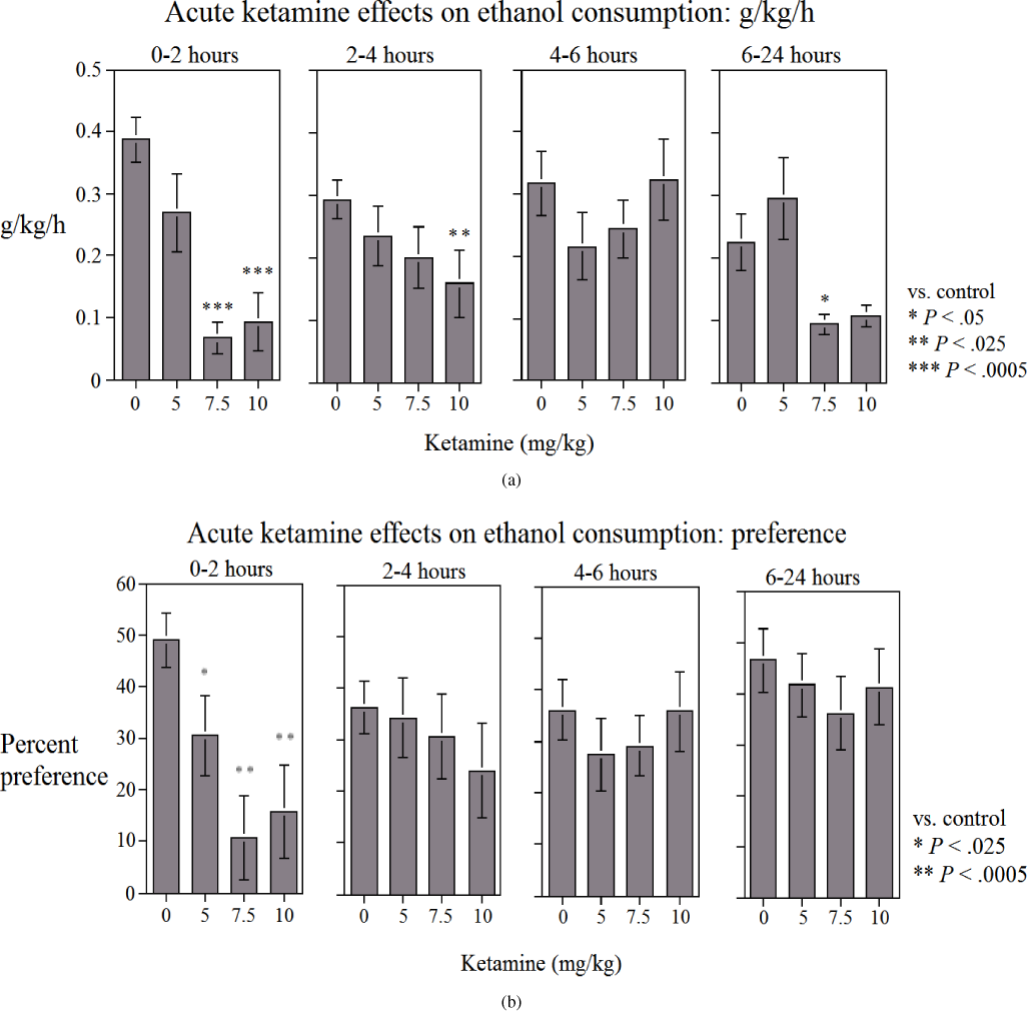
Effects of various doses of ketamine on (a) alcohol consumption (g/kg/h) and (b) percent alcohol preference [alcohol volume/(alcohol volume + water volume) × 100], during the 24-hour test (broken into various time periods) in alcohol preferring (P) rats. Both sexes are combined. Ketamine was injected SC 15 min before alcohol exposure. Values are mean ±SEM. *N* = 8 male and 10 female rats.

Alcohol intake (g/kg/h) and percent preference for alcohol were reduced by ketamine with a significant main effect for intake (*F*(3, 48) = 7.65, *P* < .005) and preference (*F*(3, 48) = 3.86, *P* < .025), resulting in a dose-dependent decrease in alcohol consumption and alcohol preference. During the first two hours, alcohol intake was reduced by approximately 29% (*P* > .05) with 5 mg/kg ketamine, approximately 81% (*P* < .005) with 7.5 mg/kg ketamine, and approximately 77% (*P* < .005) with 10 mg/kg ketamine when considering all the animals (Figure 1(a)). Similarly, alcohol preference appeared to be reduced dose-dependently by ketamine during the 24 hours, however, the effect was most distinctly observed during the first two hours. Thus, percent preference for alcohol was reduced by approximately 39% (*P* < .025) with 5 mg/kg ketamine, approximately 80% (*P* < .005) with 7.5 mg/kg ketamine, and approximately 70% (*P* < .0005) with 10 mg/kg ketamine during the first two hours (Figure 1(b)).

### 3.2. Ketamine effects on male and female alcohol consumption and preference

**Figure 2:**
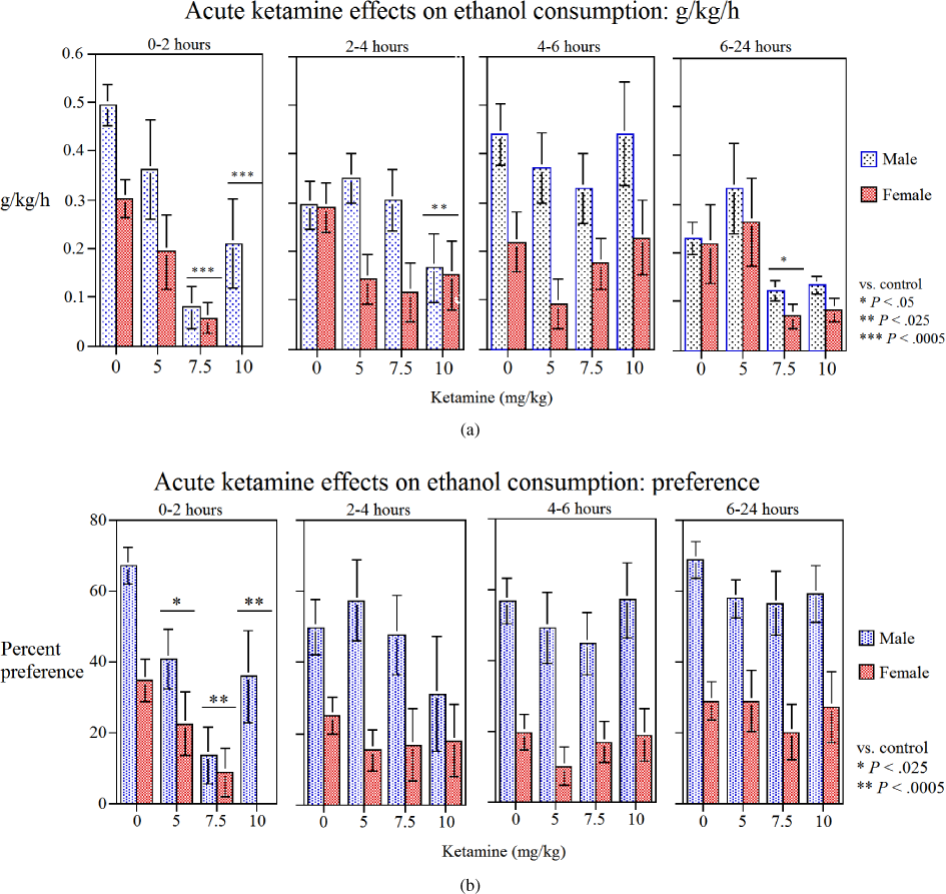
Effects of various doses of ketamine on (a) alcohol consumption (g/kg/h) and (b) percent alcohol preference [alcohol volume/(alcohol volume + water volume) × 100], during the 24-hour test (broken into various time periods) in male and female alcohol preferring (P) rats. Ketamine was injected SC 15 min before alcohol exposure. Values are mean ±SEM. *N* = 8 male and 10 female rats.

When considering the effects of ketamine on alcohol intake in male and female rats separately, there was a distinct difference between responses of males and females to this drug. There was a significant main effect of treatment × sex (*F*(1, 16) = 9.41, *P* < .01), where control male rats drank approximately (*P* < .025) 63% more compared to females in the first two hours (Figure 2(a)). In addition, during this period, ketamine at 5 mg/kg resulted in approximately 27% decrease in alcohol intake in males and approximately 33% decrease in females. These effects were not statistically significant. At 7.5 mg/kg, ketamine resulted in approximately 86% decrease in alcohol intake in males (*P* < .005) and approximately 80% decrease in females (*P* < .005). The most distinct difference between the sexes was with 10 mg/kg ketamine, where there was approximately 58% decrease in alcohol intake in males (*P* < .005) and almost 100% decrease in females (*P* < .005) as practically no response could be recorded in this group during the first two hours (Figure 2(a)).

Similarly, when considering the effects of ketamine on alcohol preference in male and female rats separately, there was a distinct difference between responses of males and females to this drug. There was a significant main effect of treatment × sex (*F*(1.16) = 20.67, *P* < .005). During the first two hours specifically, control male rats showed approximately 90% (*P* < .025) more preference for alcohol compared to females (Figure 2(b)). In addition, ketamine at 5 mg/kg resulted in approximately 40% decrease (*P* < .025) in alcohol preference in males and approximately 35% decrease (*P* < .025) in females during this time period. At 7.5 mg/kg, ketamine resulted in approximately 80% decrease in alcohol preference in males (*P* < .005) and approximately 75% decrease in females (*P* < .005). Here also, the most distinct difference between the sexes was with 10 mg/kg ketamine, where there was approximately 48% decrease in males (*P* < .005) and almost 100% decrease in females (*P* < .005) as practically no response could be recorded in this group during the first two hours (Figure 2(b)).

## 4. Discussion

The results of this study indicate that antagonism of NMDA glutamatergic receptor by ketamine may reduce alcohol intake in animal models reflective of addiction to alcohol. Although use of ketamine per se for actual clinical intervention in alcoholism or AUDs may appear controversial in the face of ketamine’s abuse liability, our findings do provide a strong basis for pursuit of investigations in glutamatergic manipulation in alcohol addiction. Indeed, numerous recent reports support such an approach in alcoholism or AUDs [6, 7, 8]. Ample evidence points to adaptive changes in glutamatergic system following chronic alcohol exposure, and involvement of glutamate signaling in alcohol’s intoxicating and rewarding effects. Specifically, it is hypothesized that chronic alcohol abuse produces a hyperglutamatergic state and that blocking NMDA and AMPA receptors (e.g., GluN2B, GluA3) can reduce alcohol consumption in rodents, but again, side-effects may limit such therapeutic approaches [7]. As mentioned before, acamprosate, a currently used drug to combat alcoholism or AUDs, is postulated to act, at least partially, via inhibition of NMDA receptors [1, 2]. In addition, a role for glutamate transmission in regulation of the mesolimbic system that is intimately involved in the rewarding effects of many drugs of abuse, including alcohol, has been indicated [6]. The mesolimbic dopaminergic system, implicated in feeling of reward or pleasure, consists of the VTA and its connection to the NAcc, stimulation of which leads to dopamine release in the NAcc [12, 13]. Alcohol has been shown to activate this system [12, 13]. Interestingly, drugs which upregulate glutamate transporter 1 (GLT1) expression in mesocorticolimbic circuit can reduce alcohol intake in genetic animal models of alcoholism by normalizing the hyperglutamatergic state in this circuit [8, 14].

It should be noted that ketamine is currently used as a dissociative anesthetic in humans and animals. It is referred to as a “dissociative anesthetic” because it makes patients feel detached from their pain and environment. It is abused for its ability to produce dissociative sensations and hallucinations. These effects are invariably associated with relatively high dose of ketamine, which might involve interaction with other neurotransmitter systems including the opioid, cholinergic, dopaminergic, and adenosine receptors as well as inflammatory cytokines [15, 16, 17]. Recently, it has become evident that subanesthetic doses of ketamine can have a rapid and long lasting antidepressant effect in various animal models [18] as well as in clinical trials. Indeed, there are some centers in the United States that use ketamine for treatment-resistant depression as well as suicidal ideation [19]. Interestingly, the antidepressant effects of ketamine were shown to be blocked by NBQX, an AMPA/kainate receptor antagonist [20]. Moreover, of relevance to our study is the finding that a kainate, but not AMPA antagonist, was effective in reducing alcohol intake [21]. In the same vein, studies need to be performed on chronic effects of such drugs as well as duration of such effects.

Another important outcome of our studies was the finding of differential sensitivity of the two sexes to the effects of ketamine in alcohol intake/preference. As shown in Figure 2, female rats were more sensitive to the attenuation effects of ketamine on alcohol intake and alcohol preference. Gender influences on drug addiction in humans [9, 22, 23, 24] and animals [9, 25, 26, 27] as well as differential response to treatments in the two sexes are well established. Differential sensitivity to the effects of alcohol in males versus females is also amply evident [6, 9, 28]. Specifically, in regard to interaction with glutamatergic system, alcohol has been shown to differentially affect basal glutamate levels in male compared to female rats [6]. Hence, future studies have to consider the impact of gender as well as age on feasibility of glutamatergic intervention in alcoholism or AUDs. It is also of relevance to note that differential sensitivity between two sexes to ketamine, in terms of locomotor sensitization (females being more sensitive than males), has been recently reported [29]. Moreover, although the high dose of ketamine is sedative, the low doses of ketamine used in our study do not appear to result in any sedation [18, 29, 30].

In summary, our results suggest that glutamatergic receptor manipulations may be of therapeutic potential in addiction to alcohol and that different sexes may respond differentially to such treatments.
